# Cardiovascular risk and endothelial function in people living with HIV/AIDS: design of the multi-site, longitudinal EndoAfrica study in the Western Cape Province of South Africa

**DOI:** 10.1186/s12879-016-2158-y

**Published:** 2017-01-07

**Authors:** Hans Strijdom, Patrick De Boever, Gerhard Walzl, M. Faadiel Essop, Tim S. Nawrot, Ingrid Webster, Corli Westcott, Nyiko Mashele, Frans Everson, Stephanus T. Malherbe, Kim Stanley, Harald H. Kessler, Evelyn Stelzl, Nandu Goswami

**Affiliations:** 1 Division of Medical Physiology, Faculty of Medicine and Health Sciences, Stellenbosch University, PO Box 241, Cape Town, 8000 South Africa; 2 Environmental Risk and Health Unit, Flemish Institute for Technological Research (VITO), Boeretang 200, 2400 Mol, Belgium; 3 Centre for Environmental Studies, Hasselt University, Agoralaan, 3590 Diepenbeek, Belgium; 4 Division of Molecular Biology and Human Genetics, DST/NRF Centre of Excellence for Biomedical Tuberculosis Research and SAMRC Centre for Tuberculosis Research, Faculty of Medicine and Health Sciences, Stellenbosch University, PO Box 241, Cape Town, 8000 South Africa; 5 Cardio-Metabolic Research Group (CMRG), Department of Physiological Sciences, Faculty of Science, Stellenbosch University, Stellenbosch, 7600 South Africa; 6 Department of Public Health and Primary Care, Leuven University, Kapucijnenvoer 35, 3000 Leuven, Belgium; 7 Institute of Hygiene, Microbiology and Environmental Medicine, Medical University of Graz, Universitätsplatz 4, 8010 Graz, Austria; 8 Gravitational Physiology and Medicine Research Unit, Institute of Physiology, Medical University of Graz, Harrachgasse 21/V, 8010 Graz, Austria

**Keywords:** HIV, Antiretroviral therapy, Cardiovascular risk factors, Vascular endothelial function, South Africa

## Abstract

**Background:**

There is growing evidence of an interaction between HIV-infection, anti-retroviral therapy (ART) and cardiovascular diseases (CVD). Epidemiological studies in Europe and North America have been observing a shift towards an increased incidence of coronary heart disease and acute myocardial infarctions in HIV-infected populations compared to the general population even after adjusting for traditional cardiovascular risk factors. Despite South Africa (and sub-Saharan Africa, SSA) being regarded as the epicentre of the global HIV epidemic, very little is known about the prevalence of cardiovascular risk factors and precursors of vascular disease in HIV-infected populations in this region. The knowledge gap is further widened by the paucity of data from prospective studies. We present the rationale, objectives and key methodological features of the EndoAfrica study, which aims to determine whether HIV-infection and ART are associated with altered cardiovascular risk and changes in vascular endothelial structure and function in adults living in the Western Cape Province of South Africa.

**Methods:**

In this longitudinal study, comprehensive cardiovascular assessments of HIV-negative and HIV-positive (with and without ART) study participants are performed by clinical and biochemical screening for traditional cardiovascular risk factors and biomarkers of CVD. Vascular and endothelial function is determined by brachial artery flow-mediated dilatation (FMD), carotid-intima-thickness (IMT) measurements and quantitative retinal blood vessel analyses, complemented by vascular endothelial biomarker assays. Finally, we aim to statistically determine whether HIV-infection and/or ART are associated with increased cardiovascular risk and vascular endothelial dysfunction, and determine whether there is progression/regression in these endpoints 18 months after the baseline assessments.

**Discussion:**

The EndoAfrica study provides a unique opportunity to recruit a cohort of HIV-infected patients and HIV-negative controls who will be comprehensively and longitudinally assessed for cardiovascular risk and disease profile with vascular endothelial function as a potentially important intermediate cardiovascular phenotype. To our knowledge, it is the first time that such a systematic study has been established in the context of SSA and South Africa.

## Background

There is growing evidence of an interplay between HIV-infection, anti-retroviral therapy (ART) and cardiovascular diseases (CVD) [1]. The trends with regards to the nature of HIV-related CVD have evolved since the introduction of highly active ART (HAART). HIV-associated cardiomyopathy was one of the first CVD conditions described in HIV-infected study populations [2–5], with systolic dysfunction particularly prevalent in the pre-HAART era. Since the introduction of HAART, epidemiological studies in Europe and North America have been observing a shift towards an increased incidence of coronary heart disease in HIV-infected populations compared to the general population [1, 6–8]. For example, a large prospective cohort study in the USA with ~82,000 participants showed that HIV-infected participants had a 50% increased risk of acute myocardial infarction even after adjusting for traditional cardiovascular risk factors [9].

Sub-Saharan Africa (SSA) is home to approximately 26-million (70%) of the world’s HIV-infected population [10]. As a result, SSA has witnessed the roll-out of massive ART programmes in recent years. For example, South Africa has the world’s largest Government-sponsored programme that reached approximately 3.1-million people in 2015 [11]. In addition to the HIV/AIDS epidemic, many countries in SSA are subjected to a rapidly expanding non-communicable diseases epidemic, characterised by, among others, a high prevalence of traditional cardiovascular risk factors and disease [12]. In fact, it has been reported that 80% of all cardiovascular-related deaths globally occur in low- to middle income countries [13]. In South Africa, the growing prevalence of traditional cardiovascular risk factors such as hypertension, obesity and diabetes mellitus has been described as a “time bomb” [14], and the multiple burden of communicable and non-communicable diseases a “collision of epidemics” [15]. Surprisingly, the impact of high HIV-infection rates and HAART on the increasing prevalence of CVD in SSA has received relatively little research attention and the knowledge gap is further widened by the paucity of data from longitudinal studies.

Based on the limited epidemiological data available, pulmonary hypertension, cardiomyopathy and tuberculous pericarditis are reported to be the most common cardiovascular-related pathologies associated with HIV-infection in SSA populations [12, 16]. The incidence of coronary artery disease in SSA is relatively low, not only in the general population [17], but seemingly also in people affected by HIV-infection [18]. However, there is reason to believe that the current predominantly “pre-HAART” cardiovascular disease profile of sub-Saharan Africans living with HIV/AIDS may change. Taking into account the increase in coronary artery disease observed in HIV-infected populations from Europe and USA since the introduction of HAART, it is conceivable that the full cardiovascular health impact of the HAART roll-out programmes initiated in SSA in recent years has not yet been felt [12]. In this regard, data are emerging from countries such as Kenya and South Africa showing a higher incidence of traditional (predominantly pro-atherogenic) cardiovascular risk factors, such as hypertension, obesity and dyslipidaemia in HIV-infected participants [19–21].

Vascular endothelial activation and endothelial dysfunction are now recognized as early markers of HIV-related CVD [1, 22, 23], and recent data from South Africa confirmed that HIV-infection was associated with increased inflammation-induced endothelial injury, endothelial activation, and endothelial dysfunction [24, 25]. Vascular endothelial changes occur early in the cardiovascular disease process, and endothelial dysfunction is widely recognized as a marker of the net effects of cardiovascular risk factors as well as future cardiovascular events [26]. Hence, the inclusion of vascular endothelial function assessment as a surrogate marker of CVD in clinical research in SSA populations has recently been advocated [27, 28].

### Problem statement

The HIV/AIDS epidemic in SSA appears to be on a collision course with the rapidly increasing burden of cardiovascular risk factors and disease. However, the scope and nature of the cardiovascular risk and disease profile, including early vascular and endothelial changes, of HIV-infected populations in SSA remains unclear. Hence, the need for epidemiological studies to address this knowledge gap is great. Studies should screen for traditional endpoints of cardiovascular risk in HIV/AIDS-exposed populations, and also incorporate endpoints that provide early (preclinical) and predictive information on cardiovascular risk and future events, such as vascular endothelial function measurements.

The EndoAfrica study was established to provide a comprehensive and repeated assessment of the cardiovascular risk and disease profile in South African adults living with HIV/AIDS. The study includes clinical screening and extensive biomarker analysis of vascular endothelial function. To our knowledge, it is the first time that such a systematic study has been established in the context of SSA and South Africa.

## Methods/design

### Aim and objectives

The overarching aim of the EndoAfrica study is to determine whether HIV-infection and ART are associated with altered cardiovascular risk and changes in vascular endothelial structure and function in adults living in the Western Cape Province of South Africa. The following objectives have been defined:

1. To perform comprehensive cardiovascular assessments of HIV-negative and HIV-positive (with and without ART) study participants by clinical and biochemical screening for traditional cardiovascular risk factors (overweight/obesity, smoking, hypertension, diabetes mellitus, dyslipidaemia) and biomarkers of CVD;
2. To perform assessments of vascular and endothelial function by ultrasound-based flow-mediated dilatation (FMD) measurements of the brachial artery, carotid-intima-thickness (IMT) measurements, quantitative retinal blood vessel analysis, and vascular endothelial biomarker analyses;
3. To determine statistically whether HIV-infection and/or ART are associated with increased cardiovascular risk and vascular endothelial dysfunction, and determine whether there is progression/regression in these endpoints 18 months after the baseline assessments.

### Study design and setting

EndoAfrica uses a repeated measures design, and study participants undergo baseline and 18 months’ follow-up assessments. Assessments include a personal interview and completion of a comprehensive health questionnaire, standard anthropometric measurements, a cardiovascular system-focused physical examination, blood and urine collection for biochemical and biomarker analyses, ultrasound-based vascular and endothelial assessments (FMD and carotid IMT), and fundus imaging for retinal blood vessel analysis. The study allows the investigators to monitor the progression or regression of the major study endpoints in participants over a 18-month period. EndoAfrica, which commenced officially in April 2015, has received ethics approval from the relevant body at Stellenbosch University (see later) and is conducted in Cape Town and Worcester in the Western Cape Province of South Africa.

### Participant recruitment and study groups

The study population mainly consists of patients visiting primary health care clinics. Participants are recruited by qualified and experienced research nurses at community health care centres and/or HIV clinics in Cape Town (Elsies River, Adriaanse, Fisantekraal, Uitsig, Durbanville and Ravensmead), and the local community health centre in Worcester based on pre-determined inclusion and exclusion criteria. For inclusion into the study, participants must be 18 years or older, and female participants must not be pregnant or less than 3 months post-partum. Participants will be excluded from the study if they are younger than 18 years, pregnant, or less than 3 months post-partum. Participants who qualify for inclusion are invited to take part in the study and requested to sign an informed consent form.

**Fig. 1:**
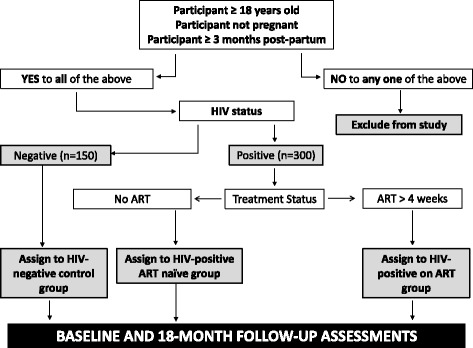
Recruitment protocol and study groups

Once enrolled, participants with unknown HIV status are counselled for HIV testing and screened for HIV-infection by performing a rapid HIV test (SD Bioline HIV 1/2 3.0 immunochromatographic test kit; Standard Diagnostics, Republic of Korea). If the test result is negative, the participant is assigned to the HIV-negative control group. Participants with confirmed HIV-infection are assigned to the HIV-positive group, which is further divided into two sub-groups based on the ART status of participants. Those participants not receiving ART are assigned to the “HIV-positive ART naïve” sub-group. Current South African ART guidelines recommend that all newly diagnosed HIV-infected patients be placed on ART regardless of their CD4 count. In view of this, one has to realistically assume that the majority of participants in this sub-group will likely commence with ART soon after recruitment. This provides a unique opportunity to assess cardiovascular risk and early endothelial changes in both the untreated (baseline) and treated (18 months’ follow-up) state in the same participants. Therefore, the effects of ART can be assessed in individuals who serve as their own untreated controls. The second sub-group consists of HIV-infected participants who have been on ART for at least four weeks (“HIV-positive on ART”). The CD4 count and viral load of the HIV-positive participants will be determined at baseline and 18 months’ follow-up. All participants are required to fast from 22 h00 the night before the assessments. They are also asked to refrain from smoking, drinking coffee or doing exercise for 4–6 h prior to the study, and female participants should report the phase of their menstrual cycle. Figure 1 depicts the study design, inclusion/exclusion criteria and study groups.

### Assessments: clinical and laboratory investigations

#### Health questionnaire, anthropometry, cardiovascular measurements, blood and urine chemistry

General cardiovascular health information is obtained from questionnaires and personal interviews (including family and personal history of cardiovascular disease, medication, smoking, and alcohol consumption). In addition, participants are screened for cardiovascular risk factors by anthropometric measurements, heart rate and blood pressure measurements, and blood and urine chemistry analyses. The anthropometric assessments, *viz.* body-mass-index (BMI), waist circumference and waist-to-hip ratio, are performed according to international standards [29]. Heart rate and brachial systolic and diastolic blood pressure of the left arm are measured at three different occasions, 2–5 min apart, with an Omron M6 automatic digital blood pressure monitor (Omron Healthcare, Kyoto, Japan). Blood and early morning mid-stream urine samples are obtained by the research nurse from each participant. Samples are transported to the National Health Laboratory Service (NHLS) laboratory in the Tygerberg Academic Hospital, adjacent to the Faculty of Medicine and Health Sciences of Stellenbosch University. Here, the following markers of cardiovascular risk are measured: (i) Lipids (total cholesterol, LDL-cholesterol, HDL-cholesterol and triglycerides), (ii) glucose, (iii) glycated haemoglobin (HbA1c), (iv) creatinine, (v) gamma-glutamyl transferase (GGT), (vi) highly sensitive C-reactive protein (hs-CRP), (vii) haemoglobin, and (viii) urine albumin : creatinine ratio. The measurements are performed according to NHLS approved standard analytical protocols. Collectively, results from the above interviews and investigations allow the researchers to screen for the following cardiovascular risk factors: smoking, alcohol consumption, overweight/obesity, hypertension, diabetes mellitus, dyslipidaemia, renal impairment, inflammation and liver disease.

#### Flow mediated dilation (FMD)

FMD is regarded as the gold standard of noninvasive vascular endothelial function assessment in the research setting [26, 30]. Several studies have been performed in which FMD measurement of vascular endothelial function was validated in terms of prognostic ability as well as successful disease progression/regression monitoring in clinical trials [30]. FMD has also previously been shown to be a marker of effect in populations living with HIV/AIDS [31, 32].

The FMD procedure used in the EndoAfrica study is based on a previously described protocol [30], and performed by research assistants who have been trained by specialists in the field. According to the protocol, the participant rests in the supine position for 10 min prior to the procedure. A longitudinal image of the right brachial artery is obtained in B-mode with an Esaote MyLab^TM^Five ultrasound and 12 MHz linear probe (Esaote, Italy). The probe is stabilised on the upper-arm using a single axis precision probe holder from SMT Medical (Wuerzburg, Germany) that allows for micro-metric adjustments. Doppler mode is used to visualise and locate the brachial artery with pulse repetition frequency (PRF) set at 6.7 kHz. Once the artery is properly visualised, depth is increased to 3 cm before switching to pulse wave mode. In pulse wave mode, the angle of insonation is set to +60°. Brachial artery diameter and shear rate measures are recorded continuously with FMD Studio and Cardiovascular Suite version 2.8.1 software (Quipu, Italy), which makes use of automatic edge detection technology. The protocol starts with 1 min baseline recording at rest. Following this, a blood pressure cuff around the fore-arm is inflated to 50 mmHg above the systolic blood pressure value and remains inflated for 5 min, this is called the ischaemic phase. After 5 min, the cuff is deflated resulting in hyperaemia and increased shear rate inducing endothelium-dependent dilatation of the brachial artery, followed by the recovery phase (total recording time: 3 min). At the end of the procedure, the software calculates baseline artery diameter, maximum artery diameter, recovery artery diameter, FMD % (difference between maximum and baseline diameter expressed as %), baseline shear rate and maximum shear rate.

#### Carotid intima-media thickness (IMT)

Carotid IMT is an important biomarker of arterial wall thickening and subclinical and clinical atherosclerosis [33, 34]. Increases in IMT are associated with both prevalent and incident cardiovascular morbidity and mortality, including coronary heart disease [35]. IMT measurements will follow protocols and guidelines as described previously [36]. The participants are in the supine position with the head tilted 30° to the left for assessment of the right carotid artery, and 30° to the right for assessment of the left carotid artery with the Esaote MyLab^TM^Five ultrasound and 12 MHz linear probe (Esaote, Italy). Once the carotid artery bifurcation is visualised manually in b-mode, a 10 mm straight arterial segment is selected in the common carotid artery proximal to the bifurcation bulb. The segment must be free of atherosclerotic plaque and the lumen-intima and media-adventitia interfaces must be clearly defined. Dedicated Quality Intima-Media Thickness (QIMT) software is used for real-time radio frequency detection of the IMT, which automatically measures the intima-media thickness. The software additionally calculates the median ± standard deviation IMT values during several cardiac cycles [37].

#### Retinal vessel analyses

Retinal image analysis is an unobtrusive procedure for visualizing the microcirculation. The microcirculation consists of blood vessels less than ~150 μm and includes smallest resistance arteries, arterioles, capillaries, and venules. These vessels make up a large part of the circulatory system and play an important role in maintaining cardiovascular health. There is substantial evidence in favour of a correlation between retinal vascular changes and coronary heart disease [38, 39]. The ratio between the diameter of retinal arteries and retinal veins (A/V) has been shown to be a sensitive proxy to reflect hypertension and atherosclerosis [40]. A narrowing of the arteries and widening of the veins, leading to a decreased A/V ratio, corroborates with risk of stroke and myocardial infarction [41]. Quantitative features extracted from retinal images have been proven useful for identifying microvascular changes with predictive power in the field of cardiovascular disease development [42].

Fundus images are captured with a non-mydriatic digital retinal camera (Canon CR-2 camera, Canon Europa NV, The Netherlands). The retinal vessel dimensions, vascular tree and microvascular state are analysed with semi-automated IFLEXIS software developed by VITO (Belgium). Analysis of retinal images are undertaken by a trained grader, masked to participant characteristics. The grader performs the vessel measurements on the optic disc–centred image of both the right and left eye; however, the data of the right eye are used for most participants and left eye data only when the right eye images are ungradable. The software allows calculating vessels widths and pattern features. For the calculation of vessel width, the largest six arterioles and venules coursing through a zone between 0.5 and 1 disc diameter from the optic disc margin are measured. Estimates are summarized as central arteriolar equivalent (CRAE) and central retinal venular equivalent (CRVE), representing the average diameter of arterioles and venules of the eye, respectively [43]. For the calculation of pattern features such as tortuosity, fractal analysis, and lacunarity, all blood vessels coursing through a zone of 2x disc diameter from the optic disc margin are measured. An overview of the different retinal features that are calculated are also reported in a recent study by Prabhakar and colleagues [44].

#### Serum biomarker analyses

An array of serum biomarkers of vascular endothelial dysfunction will be measured to complement the clinical measurements. The following biomarkers have previously been shown to be associated with vascular endothelial dysfunction, and will be measured in this study: vascular cellular adhesion molecule-1 (VCAM-1), intercellular adhesion molecule-1 (ICAM-1), e-selectin, p-selectin, platelet activating inhibitor-1 (PAI-1), tumor necrosis factor-alpha (TNF-α), vascular endothelial growth factor (VEGF) and von Willebrand Factor [22]. These markers will be measured by Luminex technology (Luminex Bio-Plex 200 system) in the Division of Molecular Biology and Human Genetics, Faculty of Medicine and Health Sciences, Stellenbosch University. The Luminex cytokine/chemokine/protein assays are sandwich immunoassays that are performed in 96-well filter plates, similar to sandwich ELISA assays, and can analyse up to 100 different host markers in each well.

**Fig. 2:**
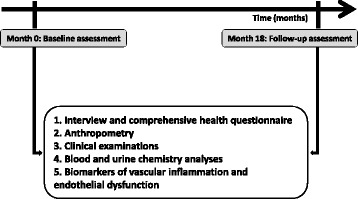
Clinical and biochemical assessments of study participants at baseline and 18 months’ follow-up

Figure 2 shows the baseline and 18 months’ follow-up assessments.

### Data collection, management and statistical analysis

All data generated from the health questionnaires, clinical assessments and biochemical investigations will be collected and managed using REDCap electronic data capture tools hosted at the Department of Biomedical Sciences, Stellenbosch University [45]. REDCap (Research Electronic Data Capture) is a secure, web-based application designed to support data capture for research studies.

Data are statistically analyzed in conjunction with the Biostatistics Unit in the Centre for Evidence-based Health Care, Stellenbosch University. Descriptive statistical models (ANOVA, Chi Square) will be used for inter-group comparisons, and associations between independent and dependent variables will be tested by multivariate regression analysis models, adjusting for age, gender, ethnicity, smoking, alcohol consumption, medication and BMI. For longitudinal changes within the same individual, a repeated measures model approach will be employed.

A literature search was undertaken with the assistance of the Biostatistics Unit (Faculty of Medicine and Health Sciences, Stellenbosch University), and according to Donald et al. [46], a case sample size of between *n* = 100–400 over a 3 months’ follow-up period was sufficiently powerful to secure endothelial function measurement reproducibility and statistical significance (1–2% effect size; 80% statistical power and 5% significance). In our own pilot studies performed in 2015 and early 2016, several cardiovascular risk factors (BMI, HDL-cholesterol, blood pressure) were significantly different between HIV-negative control and HIV-positive participants, and HIV-infection was associated with increased triglyceride levels and highly sensitive CRP values (HIV-positive sample size: *n* = 110). We are therefore confident that the proposed sample sizes will be sufficient to secure sufficient statistical power.

At the time of writing, EndoAfrica has recruited a total of 231 participants, of whom 60 are HIV-negative control and 171 are HIV-infected (ART naïve: *n* = 38). The characteristics of the cohort at this stage show a mean age of 39.23 ± 9.98 years, predominantly female participants (74.8%) with a high smoking prevalence (60.2%). The study is on-going, and provisional completion date is December 2018.

**Fig. 3:**
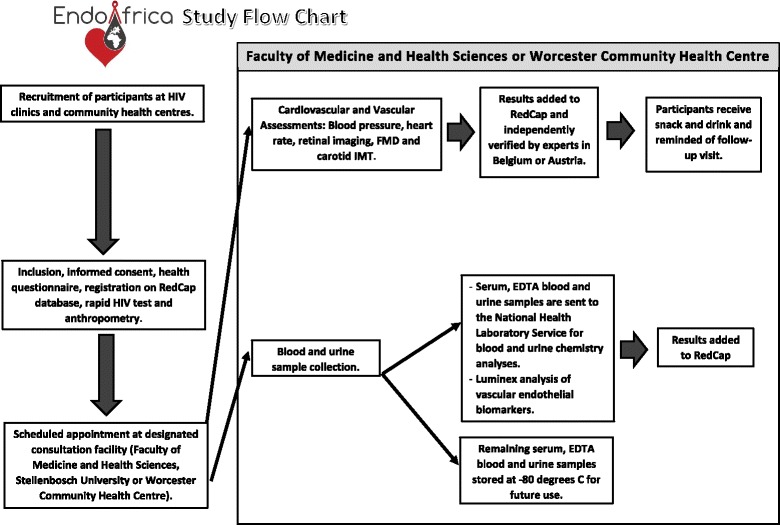
Study overview and operating procedures. FMHS: Faculty of Medicine and Health Sciences; FMD: flow-mediated dilatation; IMT: intima-media thickness; NHLS: National Health Laboratory Service

See Fig. 3 for a flow chart showing an overview of the study and operating procedures.

## Discussion

The EndoAfrica study will generate novel data in the South African (and SSA) context. We are not aware of any previous longitudinal studies in sub-Saharan Africa undertaken to comprehensively assess cardiovascular risk and vascular endothelial changes in adults living with and without HIV/AIDS. The repeated measures design of EndoAfrica will provide valuable insights into the progression or regression of markers of cardiovascular risk and disease in HIV-infected subjects. This information can help to develop guidelines for the management of cardiovascular health in patients with HIV-infection in the local health care system. The findings of the EndoAfrica study will furthermore contribute to the current global knowledge base on HIV and cardiovascular disease, which is largely devoid of data from SSA.

The vascular and endothelial endpoints of the EndoAfrica study will not only generate novel research data in the South African context, but also have the potential to predict future cardiovascular events. With a few exceptions, very little data are available on endothelial dysfunction in adult SSA populations in general, and in HIV-infected populations in particular. The fact that three independent, non-invasive imaging techniques, complemented by the measurement of a battery of serum biomarkers will be employed, can be regarded as a strength of the study.

### Possible limitations

The ethnic composition of the Western Cape Province of South Africa, and hence of our study cohort, may limit the possibility to extrapolate the findings to the rest of the population. The majority of the population in Cape Town and Worcester is of mixed ancestry, whilst the majority of HIV-infected South Africans are ethnic black Africans. The researchers will endeavour to recruit as many black study participants as possible, and steps to incorporate recruitment sites in other provinces where more black participants can be included are currently undertaken. In addition, the follow-up period of 18 months may be too short to sufficiently determine the progression of cardiovascular risk and vascular changes. However, there have been previous prospective studies employing similar or shorter follow-up periods that could demonstrate disease progression or regression (e.g. increased carotid IMT progression in HIV-infected participants after 12 months [47], and regression of endothelial dysfunction biomarkers after median 7.2 months ART [48]). The 18-month follow-up period was chosen in view of the total project duration of 3 years. The EndoAfrica research team is now building capacity and developing sustainable collaboration with the aim to extend the follow-up period of the longitudinal cohort beyond the lifetime of the initial project.

Recently, the South African Government announced that all HIV-infected patients will immediately commence with ART compared to the previous guidelines of initiating ART at a CD4 count cut-off of 500 cells/μL. This may limit our ability to recruit ART naïve participants. Finally, the researchers need to take cognizance of the fact that the FMD and IMT procedures have certain limitations in terms of operator dependency and inter-operator variability. Ensuring that the same vascular regions are assessed in each participant at the 18 months’ follow-up visit is also a potential challenge. In order to address this as far as possible, we will limit the number of operators in the team, subject them to intensive and expert training, and employ rigorous verification and quality control measures by independent experts in this field.

## Conclusion

EndoAfrica is an important and relevant study in the context of cardiovascular risk and vascular endothelial dysfunction in HIV-infection. South Africa (and sub-Saharan Africa) is the epicentre of the global HIV epidemic, yet very little is known about the prevalence of cardiovascular risk factors and early vascular disease in HIV-infected populations in this region. The EndoAfrica study addresses this knowledge gap.
